# Impact of artifact reduction on dimensional accuracy of sealing materials for furcal perforations in CBCT

**DOI:** 10.1590/1807-3107bor-2025.vol39.097

**Published:** 2025-10-20

**Authors:** Caroline Cristina BORGES, Jardel Francisco MAZZI-CHAVES, Jesus Djalma PÉCORA, Bruno Correa AZEVEDO, Giampiero ROSSI-FEDELE, Mike BUENO, Manoel Damião SOUSA-NETO, Carlos ESTRELA

**Affiliations:** (a) Universidade de São Paulo – USP, Dental School of Ribeirão Preto, Department of Restorative Dentistry, Ribeirão Preto, SP, Brazil.; (b) University of Louisville, Department of Diagnosis and Oral Health, Louisville, KY, USA.; (c) University of Adelaide, Adelaide Dental School, Department, Adelaide, SA, Australia.; (d) Universidade Federal de Goiás – UFG, Dental School, Department of Endodontics, Goiás, GO, Brazil.

**Keywords:** Artifacts, Cone-Beam Computed Tomography, Root Canal Filling Materials, Image Enhancement

## Abstract

This study aimed to evaluate the impact of the blooming artifact reduction (BAR) algorithm on the measurement of various sealing materials (Biodentine, MTA ProRoot, MTAFlow, and amalgam) for furcal perforation repair using cone-beam computed tomography (CBCT). A total of 105 extracted human mandibular first and second molars were prepared and root-filled. Furcal perforations were created, and the specimens were randomly organized into five groups based on the sealing material used: (1) Biodentine, (2) MTA ProRoot, (3) MTAFlow, (4) Amalgam, and (5) Control (no sealing material). After setting, the specimens were embedded in wax, and the crowns were sectioned to measure the diameter of the sealing materials using a digital micrometer. CBCT scans were acquired using the Prexion 3D Elite® CT scanner. The dimensional measurements of sealing materials on the CBCT images was measured using DICOM files and the e-Vol DX software measurement tool, configured to thousandths of a millimeter. BAR algorithms were applied as follows: BAR 3 for the control group, Biodentine, and MTAFlow; BAR 2 for MTA ProRoot; and BAR 1 for amalgam. Statistical analysis was performed using ANOVA and Tukey’s test with a significance level of 5%. No significant differences were observed between the diameters of sealing materials measured using the micrometer and those obtained from CBCT images processed with BAR algorithms (Biodentine, MTA ProRoot, MTAFlow, and amalgam). The BAR algorithm in the e-Vol DX software preserved the dimensional accuracy of CBCT images for the tested sealing materials.

## Introduction

Furcal perforation involves the direct communication between the pulp chamber and the adjacent periradicular tissues in multirooted teeth. Pathological processes, such as root resorption or dental caries, may cause perforation or they can be iatrogenic, resulting from operator error during access cavity preparation, root canal preparation, or the creation of space for intraradicular post placement. This complication can lead to periodontal defects or, in severe cases, tooth loss.^1^

Clinical and radiographic evaluations are essential for diagnosing furcal perforations. Persistent bleeding during access to or preparation of a vital tooth, even after complete removal of the coronal pulp, often indicates the presence of a furcal perforation. In pulpless teeth, bleeding during instrumentation can also suggest furcal perforation. Periapical radiographs, a routine diagnostic tool, may reveal a radiolucency indicative of communication between the furcation area and the periodontal space in cases of perforation.^1,2^

While these examinations are useful, diagnosing furcal perforations based solely on two-dimensional radiographic images can be challenging. Overlapping anatomical structures and image distortion often obscure the visualization of small perforations, particularly those with reduced dimensions.^2-4^ The precise assessment of sealing material thickness is essential for evaluating the adequacy of perforation repair, ensuring marginal adaptation, and guiding decisions on reintervention or prosthetic rehabilitation.^2-4^ Cone-beam computed tomography (CBCT) provides a three-dimensional assessment, introducing new parameters to aid in diagnosis and treatment planning.^5-8^ The presence of metallic or high-density materials during CBCT scanning, however, can generate artifacts, complicating the evaluation of furcal perforations.^6,9-11^

Artifact during CBCT scanning are attributed to the “beam hardening effect,” which results from the interaction between X-ray photons of varying energy levels and materials with high atomic numbers. Dense materials preferentially absorb low-energy photons, thereby increasing the average energy of the beam. This effect produces two primary types of artifacts: “blooming artifacts” (volumetric distortion) and “cupping artifacts” (dark bands or streaks between high-density objects).^7,10-12^

To address these limitations, several strategies have been proposed to mitigate or eliminate artifact effects during CBCT image acquisition, reconstruction, and post-processing.^11,13-16^

Conventional metal artifact reduction (MAR) tools often employ linear interpolation or iterative reconstruction techniques; however, these approaches may compromise image quality or diagnostic reliability in regions with high-density materials. The blooming artifact reduction (BAR) algorithm was developed to enhance edge detection and minimize overexposure artifacts by adjusting intensity values based on four distinct image enhancement filters. This method has shown promising results in preliminary analyses, warranting further evaluation of its performance in endodontic scenarios.^13^ Bueno et al.^13^ developed the e-Vol DX software, which includes the BAR algorithm. This algorithm, designed based on the RAW image format, preserves image quality by recovering underexposed or overexposed areas, optimizing brightness and saturation in specific regions to minimize the impact of artifacts and maintain image integrity.^13^

Following the diagnosis of furcal perforation, appropriate treatment planning is critical. The long-term prognosis of perforated teeth depends on factors such as the size and location of the perforation, the duration of exposure to contamination, and the sealing material used.^1,2^ Sealing materials should be biocompatible, promote mineralized tissue formation, and provide an effective seal. Mineral trioxide aggregate (MTA) has been widely used in clinical practice for sealing perforations because of its excellent sealing properties and biocompatibility, replacing earlier materials.^17-19^

Currently, no software specifically addresses artifact reduction for high-density perforation repair materials used in CBCT imaging. Previous studies, however, have demonstrated the effectiveness of artifact reduction algorithms developed by e-Vol DX software in other clinical contexts.^1,13,20-22^

Historically, since its introduction in the 1990s, MTA has been widely regarded as a reference material for perforation repair because of its sealing ability and biocompatibility.^17,18^ Biodentine, a tricalcium silicate-based material, was developed as a bioactive alternative to MTA with improved handling properties.^19^ CeraSeal, a premixed calcium silicate-based sealer, represents a newer generation of bioceramic materials with promising physicochemical and biological properties. Although largely obsolete in modern endodontics, amalgam was historically used because it could be easily handled and was readily available.^17,18^ The inclusion of amalgam in this study enables comparisons with more contemporary materials. Accordingly, materials with varying radiopacities and chemical compositions were selected to simulate a range of clinical scenarios and assess how material density influences CBCT artifact generation and correction. Further investigation is necessary to evaluate the application of this software for sealing materials in furcal perforations, aiming to enhance diagnostic accuracy, treatment planning, and procedural outcomes. Therefore, the present study aimed to assess the role of the BAR algorithm in measuring various sealing materials (Biodentine, MTA ProRoot, MTAFlow, and amalgam) for furcal perforations using CBCT in a laboratory model.

Accurate measurement of furcal perforations is a clinically relevant challenge in endodontic practice, as it directly affects prognosis and therapeutic decision-making. The use of CBCT combined with artifact reduction algorithms, such as the BAR algorithm in the e-Vol DX software, can support long-term clinical monitoring and enhance the reliability of follow-up evaluations. Therefore, establishing the dimensional accuracy of CBCT images processed with such algorithms is essential to ensure proper treatment planning and clinical outcome assessment.

## Methods

This ex vivo observational study followed the Strengthening the Reporting of Observational Studies in Epidemiology (STROBE) guidelines.

### Sample size calculation

Sample size was calculated based on data reported by Decurcio et al.,^23^ according to which a discrepancy of 0.1 mm between CBCT and physical measurements was considered clinically relevant. A power analysis was conducted using a paired t-test, with an alpha level of 0.05, a power of 80%, and a standard deviation of 0.13 mm. The analysis indicated that 18 specimens per group would be sufficient. To increase the reliability and minimize sampling bias, we included 21 specimens per group, totaling 105 specimens.

### Specimen selection and preparation

This study was approved by the Research Ethics Committee of the Dental School at the Federal University of Goiás) under process no. 06486919.0.0000.5083. Human mandibular molars were obtained from a human tooth bank authorized by the institutional ethics committee, and all donors provided informed consent in accordance with local regulations. The original sample consisted of 135 human mandibular molars (first and second molars). Inclusion criteria were teeth with intact pulp cavity and complete root formation. Teeth were excluded if they presented fractures (n = 7), root resorption (n = 10), extensive caries compromising furcation (n = 3), or prior endodontic treatment (n = 10). These criteria were assessed via initial periapical radiographs and visual inspection. To minimize anatomical variability, only molars with similar root lengths, canal curvatures (< 20°), and furcation anatomy were included. Specimens were randomly assigned to experimental groups using a computer-generated randomization list (Random.org). A total of 105 mandibular molars were selected. The teeth were rinsed in running water for 24 h and then had their external root surface cleaned using ultrasonic scaling (Profi II Ceramic, Dabi Atlante Ltda., Ribeirão Preto, Brazil). Periapical radiographs were taken in the mesiodistal and buccopalatal directions (Spectro X70 Eletronic®, Dabi Atlante, Ribeirão Preto, Brazil) with a digital sensor (RVG 5100®, Carestream Dental, Atlanta, USA) using the paralleling technique. Specimens were then stored in flasks containing 0.1% thymol solution until the beginning of the experiment and later labeled according to the group assignment.

The specimens were removed from 0.1% thymol and immersed in 5% sodium hypochlorite (Fitofarma, Goiânia, Brazil) for 30 min to remove the remaining organic tissue. The teeth were prepared with the ProTaper Next System (PTN, Dentsply Tulsa Dental, Tulsa, USA) and prepared using the instrument sequence from X1 to X4, following the manufacturer’s instructions. Root canal filling was performed using the lateral condensation technique with gutta-percha and AH Plus® sealer (Dentsply/Maillefer, Ballaigues, Switzerland). Although the X1–X4 system may be considered extensive for the mesial root, it was selected to ensure standardized canal preparation and to enable reproducible filling procedures, as reported in prior studies using similar anatomical conditions. The lateral condensation technique was chosen for its consistent outcomes and ease of application in laboratory settings in which multiple specimens require standardized obturation. All procedures, including specimen preparation and root canal instrumentation, were performed by a single endodontist with over 10 years of clinical and research experience.

Following preparation, the tips of a #5 clinical forceps were utilized to demarcate the cutting line, with one tip placed perpendicularly on the floor of the pulp chamber and the other tip positioned on the buccal surface, establishing the line along which the crown would be sectioned perpendicularly to its long axis, close to the floor of the pulp chamber. The crowns were sectioned perpendicularly to their long axes, close to the pulp chamber floor, under constant cooling, with a speed of 350 rpm and a weight of 75 g, on a precision cutting machine (Isomet 1000, Buehler, Lake Bluff, USA). A furcal perforation at the furcal level of mandibular molars was then performed with a 0.6 mm diameter ½ carbide surgical bur (Maillefer, Ballaigues, Switzerland) at high speed under cooling. The perforation of each specimen was made at the center of the chamber floor between the mesial and distal roots. Perforation dimensions were verified using a digital caliper by two independent examiners. Intraexaminer and interexaminer reliability were assessed (ICC > 0.92), confirming the uniformity of the created defects.

A white gutta-percha shield was placed in the furcation area to avoid extrusion of the sealing material during compaction in the perforations. To place this shield, the perforation space was blocked using #40 paper cones (Dentsply Maillefer, Ballaigues, Switzerland). The paper cone was placed in the perforation until it was securely lodged. Excess paper cone protruding through the perforation was highlighted with a marker to indicate where it should be cut once removed. After cutting, the cone was repositioned in the same location for placement of the gutta-percha shield in the furcation. Once the gutta-percha set, the paper cone was removed, and the perforations were sealed with different materials.

### Sealing of furcal perforations

The teeth were randomly organized into five groups based on the sealing materials used:

G1 - Biodentine (Septodont, St. Maur-des-Fossés, France), consisting of tricalcium silicate (>70%), dicalcium silicate (<15%), zirconium oxide (5%), calcium carbonate (>10%), iron oxides (<1%), and liquid components, including distilled water, calcium chloride accelerator (>15%), and a water-soluble polymer (polycarboxylate) as a water-reducing agent;

G2 - MTA ProRoot (Dentsply Tulsa Dental, Tulsa, Oklahoma, USA), consisting of tricalcium silicate, dicalcium silicate, tricalcium aluminate (75%), bismuth oxide (20%), and gypsum. The liquid component consisted of distilled water;

G3 - MTAFlow (Ultradent, Indaiatuba, São Paulo, Brazil), consisting of a powder containing tricalcium silicate, dicalcium silicate, calcium sulfate, silica, bismuth trioxide (as a radiopacifier), and a water-based gel with soluble silicone;

G4 – Amalgam, consisting of 70% silver, 26% tin, 5% copper, and 1% zinc. Amalgam was included in the experimental groups to serve as a historical control, representing a material traditionally used for furcal perforation sealing before the introduction of bioceramic-based alternatives.

G5 - Control: No sealing material was used.

In all groups, the sealing materials were prepared and applied following the manufacturer’s instructions. The perforations were irrigated with saline solution and dried using sterilized paper cones prior to being sealed with the respective repair cements. Materials were applied using a micro-applicator under magnification, in a single increment, following the manufacturers’ instructions. No lateral condensation was performed. Precaution was taken to avoid extrusion or voids. After sealing, the access cavities were cleaned, and the specimens were organized into seven wax discs, each containing approximately 10 specimens, to facilitate CBCT acquisitions after sealing. The specimens were stored in 100% humidity at 37 °C for 7 days to ensure setting of the sealing materials before CBCT acquisition.

### Measurement of perforation diameters using a digital micrometer

The micrometer was calibrated against a certified gauge block prior to the experiment and verified daily to ensure accuracy to ±0.001 mm. Perforation diameters were measured using a calibrated digital micrometer (Mitutoyo Europe GmbH, Neuss, Germany) with an active tip diameter of 0.3 mm. Measurements were taken in both the buccopalatal and mesiodistal directions at the widest diameter, with the aid of a 3.5x magnifying glass. All values were recorded in millimeters with precision to the thousandth decimal place.

### Cone-beam computed tomography

Images were acquired in DICOM format using a PreXion 3D Elite 13-bit CBCT scanner (PreXion Inc., San Mateo, USA). The scanner was configured to produce isotropic voxel images with a resolution of 0.100 mm in a field of view (FoV) of 52 x 56 mm and an exposure time of 33.5 seconds (90 kVp, 4 mA, filter > 2.5 mm eq. Al).

After image acquisition and volumetric reconstruction, the DICOM files were processed using e-Vol DX software (CDT Software, São José dos Campos, Brazil) installed on a PC workstation equipped with Windows 10 (Microsoft Corporation, Redmond, WA, USA), an Intel i7-8750 processor running at 4.1 GHz (Intel Corporation, Santa Clara, CA, USA), and an NVIDIA GTX 1070 8GB graphics card (NVIDIA Corporation, Santa Clara, USA). Image analysis was performed using the PreXion3D Image Analysis System (TeraRecon, Inc., Foster City, USA) on a DELL P2719H monitor (27-inch, 32-bit, 1920x1080 pixel IPS panel, DELL Inc., Eldorado do Sul, Brazil).

CBCT images were visualized and analyzed following a standardized synchronization sequence in e-Vol DX software. Individual specimen images were isolated using the “cut” tool, then oriented in anatomical planes (axial, coronal, and sagittal) so that the floor of the pulp chamber was parallel to the ground, thereby correcting parallax errors. Reference points were marked on the X, Y, and Z axes to guide and standardize measurements.

**Figure 1:**
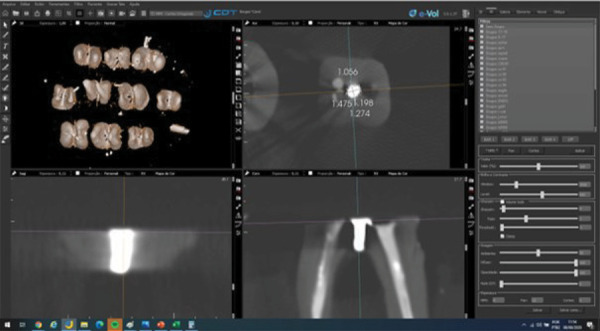
A) CBCT image of pooled specimens. B) Isolation of each specimen from one another within the acquisition process, with the help of the “cut” tool. B), C) and D) Inclination in the anatomical orientation planes (axial, coronal, and sagittal) so that the cut surface (floor of the pulp chamber view) is parallel to the ground.

Owing to image distortion caused by beam hardening and scattering artifacts from tomographic acquisition, two measurements were taken per axis. The external contour was defined as the outermost limit of the radiopaque repair material, while the internal hyperdense contour represented the denser central region, corresponding to the filling core. These contours were identified in the axial slice at the site of maximum extension of the sealing material (Fig. 1). Mean values were calculated and recorded. All measurements were performed by two oral and maxillofacial radiologists with 15 years of experience, under controlled conditions, in a silent, dimly lit room without time restrictions. Intraexaminer reliability was assessed by repeating 30% of the measurements after two weeks, with an intraclass correlation coefficient (ICC) of 0.96, indicating excellent agreement.

### Measurement using the e-Vol DX software

Original DICOM images, preserved in their original resolution, bit depth, and orientation, were imported into the e-Vol DX software. After positioning each specimen, the “color map” tool was applied.

The BAR algorithm applies four intensity filters with individual adjustments for brightness, contrast, edge enhancement, and dynamic range. The final output is an average of the results from these filtered images. While this composite approach enhances edge definition, it may introduce bias depending on the material density. To minimize this risk, identical settings and measurement criteria were used for all specimens across groups. In addition, to standardize image evaluation and avoid potential confounding factors introduced by different reconstruction conditions, the BAR algorithm was applied to all sealed groups. This tool displays grayscale images using a color spectrum, representing varying signal intensities based on the densities of different materials. Hyperdense/white areas were initially highlighted in red. These areas were analyzed using the BAR algorithm, which applies four intensity filters, each with distinct adjustments for brightness, contrast, enhancement, and dynamic range, tailored to specific repair materials. The final assessment was conducted on grayscale images to visually confirm object contours without interference from adjacent hyperdense areas.

The algorithms used were: BAR 1 for the amalgam group, BAR 2 for the MTA ProRoot group, and BAR 3 for the Biodentine and MTAFlow groups. No filter was applied to the control group. The software automatically set brightness, contrast, and enhancement parameters.

The linear measurement tool was then applied along the buccopalatal and mesiodistal axes of the perforation space. Mean values were calculated for each group.

### Statistical analysis

Data were entered into a Microsoft Excel spreadsheet and subsequently exported to the R software for statistical analysis. Symmetry of the variables was assessed using the Shapiro-Wilk test. Descriptive statistics were expressed as mean and standard deviation. The dependent variables were the buccolingual and mesiodistal dimensions of the perforation measured at the widest point. Paired Student’s t-test was used to compare measurements obtained via the digital micrometer and the e-Vol DX software. ICC was calculated to assess agreement between buccopalatal and mesiodistal measurements, and Bland-Altman plots were used to visualize agreement between the methods. To compare dimensional measurements among groups, analysis of variance (ANOVA) was performed. Tukey’s post-hoc test was applied for mesiodistal measurements (equal variances, as confirmed by Levene’s test), while the Games-Howell post-hoc test was applied for buccopalatal measurements (unequal variances). Statistical significance was set at p < 0.05.

## Results

**Figure 2:**
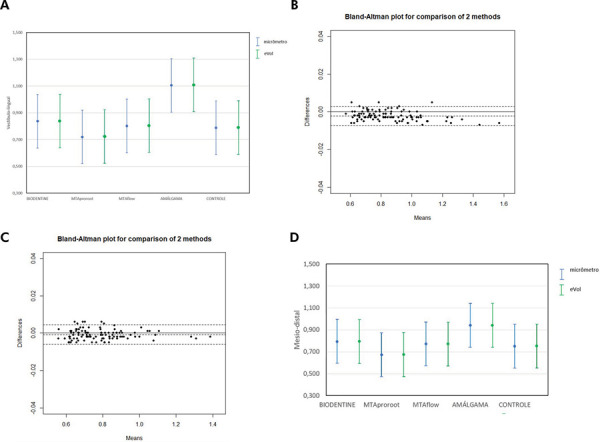
Graphs illustrating the comparison between measurements performed with a micrometer and e-Vol DX software. A) Graph showing mean and error bar comparing micrometer and e-Vol DX measurements in the buccolingual direction for each group. B) Graph showing mean and error bar comparing the micrometer and e-Vol DX measurements in the buccolingual direction for each group. C) Bland-Altman plot showing the comparison between the micrometer and the e-Vol DX measurements in the mesiodistal direction for the entire sample. D) Graph showing mean and error bar comparing the micrometer and e-Vol DX measurements in the mesiodistal direction for each group.

Figures 1 and 2 illustrate the methodology used for image analysis and measurement, as well as the agreement between the methods. Figure 1 shows representative CBCT images with specimen orientation and measurement protocol using the e-Vol DX software. Figure 2 includes graphical comparisons and Bland-Altman plots for the buccolingual and mesiodistal measurements.

The comparison of buccolingual measurements between the micrometer and the e-Vol DX software showed no statistically significant differences in the overall sample or within individual groups. The e-Vol DX software consistently recorded higher mean values (Table 1). The analysis revealed a bias of -0.002 mm, indicating that the e-Vol DX software measurements were, on average, 0.002 mm smaller than those obtained with the micrometer. The two methods agreed in 95% of the data, with differences ranging from -0.007 mm to 0.003 mm (Figure 2).

**Table 1:** Endodontic cements, presentation, composition, and setting time according to the manufacturer’s instructions.

Variables	Presentation	Setting time (min)	Composition	Manufacturer
Biodentine™	Powder/Liquid	12	Powder: Tricalcium silicate, zirconia oxide, calcium oxide, calcium carbonate, yellow and red pigment, brown iron oxide.	Septodont
			Liquid: Calcium Chloride Dihydrate, Purified Water*	Saint-Maur-des-Fossés, France
ProRoot® MTA	Powder/Liquid	165	Powder: bismuth oxide, tricalcium silicate, dicalcium silicate, calcium dialuminate and calcium sulfate. Liquid: Sterile distilled water	Dentsply
				Sirona Tulsa, USA

MTAFlow™	Powder/Gel	8,12	Powder: Tricalcium silicate Dicalcium silicate Calcium sulfate Silica Bismuth trioxide (radiopacifier). Liquid: Water Soluble silicone-based gel	Ultradent, Indaiatuba, Brasil
Amalgam Permite	Mercury alloy powder	9	Ag 56%; Sn 27,9; Cu 15,4%, In 0,5%, Zn 0,2%, Hg 47,9%	SDI, São Paulo, Brasil

In the mesiodistal measurements, the comparison between the micrometer and the e-Vol DX software also revealed no statistically significant differences in the overall sample or within the groups. The e-Vol DX software recorded higher mean values across all groups (Table 2). The methods demonstrated a bias of <0.001 mm, showing a high level of consistency. The agreement between the methods covered 95% of the data, with a range of -0.006 mm to 0.004 mm (Figure 2).

**Table 2:** Comparison table between measurements performed with the micrometer and e-Vol DX, with concordance obtained through the technique of Bland and Altman.

Micrometer			e-Vol DX software						
Group	Mean	SD	Mean	SD	p-value	Mean difference		95%CI	
								Ll	Ul
BL									
Biodentine	0.837	1.221	0.839	0.121	0.971	-0.002	-0.077	0.074	0.839
MTA ProRoot	0.720	0.101	0.723	0.102	0.925	-0.003	-0.066	0.060	0.723
MTA flow	0.802	0.125	0.804	0.127	0.972	-0.001	-0.079	0.071	0.804
Amalgam	1.105	0.119	1.109	0.197	0.949	-0.004	-0.126	0.119	1.109
Control	0.788	0.119	0.789	0.120	0.976	-0.001	-0.075	0.073	0.789
Total	0.851	0.189	0.8528	0.1334	>0.005	-0.002	-0.013	0.007	0.8528
MD									
Biodentine	0.794	0.108	0.793	0.107	0.998	0.000	-0.067	0.067	0.000
MTA ProRoot	0.673	0.070	0.675	0.070	0.901	-0.003	-0.046	0.411	-0.003
MTA flow	0.771	0.115	0.770	0.115	0.986	-0.001	-0.071	0.072	-0.001
Amalgam	0.940	0.215	0.941	0.215	0.985	-0.001	-0.135	0.132	-0.001
Control	0.751	0.100	0.752	0.100	0.985	-0.001	-0.063	0.061	-0.001
Total	0.786	0.155	0.786	0.121	>0.005	-0.001	-0.025	0.020	-0.001

Ll: lower limit; Ul: upper limit.MD: mesio-distal; SD: standard deviation; *p-value obtained through Student’s t-test for paired samples; **CI: Interval of 95% of agreement obtained using the Bland and Altman technique.

These findings confirm that the e-Vol DX software and the micrometer produce consistent measurements of furcal perforation dimensions, with only minor differences in dimensional measurements. Tables 1 and 2 summarize the results, and Figure 2 illustrates the agreement between the methods.

## Discussion

The BAR algorithm in the e-Vol DX software, designed to mitigate artifacts caused by high-density materials in CBCT images, preserved the accuracy of diameter measurements for sealing materials. These measurements showed no significant differences when compared to reference values obtained with a digital micrometer, demonstrating that the BAR algorithm effectively maintains the dimensional integrity of CBCT images. Additional studies should explore comparative performance with and without artifact reduction.

This finding is critical for accurate diagnosis and treatment planning in endodontics. Artifacts generated by high-density materials remain a significant challenge, as they distort the volumetric shape and contrast of objects, obscure structural details, and complicate diagnosis.^2,20,21^ Bioceramic sealers such as CeraSeal and MTA have distinct radiodensities that may produce varying artifact profiles. The performance of the BAR algorithm across these materials offers insight into its applicability in diverse clinical situations.

White contrast artifacts, commonly referred to as blooming artifacts, directly affect data interpretation, increase diagnostic errors, and compromise treatment planning and execution, potentially leading to tooth loss.^13,20,21^ Several studies have explored strategies to reduce or eliminate these artifacts during CBCT image acquisition, reconstruction, and post-processing.^12,21,23-28^

Although largely discontinued in contemporary endodontic practice, amalgam was included as a reference to compare the dimensional accuracy of modern sealing materials against a historically used alternative. This inclusion offers insight into the evolution of material performance in CBCT imaging and sealing outcomes. Bueno et al.^13^demonstrated that reducing bright areas in an image significantly decreases artifacts. Using the RAW image format, the BAR algorithm preserves image quality by recovering underexposed or overexposed areas, optimizing saturation and brightness, and minimizing the loss of image detail.

The findings of this study align with those of the previous research conducted by Bueno et al.,^13^ who demonstrated that the BAR algorithm effectively reduces white contrast artifacts without inducing dimensional measurements in CBCT images of intraradicular posts. Similarly, the present findings confirm that the BAR algorithm preserves dimensional accuracy for sealing materials of varying densities, providing a reliable tool for clinical applications. Conversely, other studies have reported mixed results with different artifact reduction methods. Vasconcelos et al.^24^ found that the EndoMode function in Cranex 3D software did not reduce artifacts and, in some cases, increased them. Bechara et al.^10^ observed a decrease in diagnostic accuracy when artifact reduction algorithms were applied to CBCT systems for detecting root fractures. These discrepancies highlight the importance of algorithm-specific validation, such as the consistency demonstrated by the BAR algorithm in this study.

The differences observed in the buccolingual measurements between the MTA ProRoot and amalgam groups compared to the control group can be attributed to the higher atomic number and density of these materials, which exacerbate artifact expression.^20,29,30,31^ Similarly, the differences between MTAFlow and amalgam in buccolingual measurements likely stem from the lower density of MTAFlow, which results from the addition of a gel component that reduces the percentage of radiopacifiers in its composition. These findings underscore the role of material composition in artifact expression and the importance of tailored artifact reduction strategies. Although certain discrepancies were as small as 0.002 mm, even minimal deviations may influence the perception of overfilling or underfilling in high-resolution imaging. Nonetheless, no clinical threshold or cut-off has been universally established for acceptable CBCT-based deviation in sealing thickness.

This study employed a rigorous methodology to ensure accuracy and minimize bias. Root canal instrumentation and obturation were standardized for all specimens using the same rotary system and filling technique, ensuring consistency in specimen preparation. As the focus of the study was on the measurement of sealing material dimensions in the furcal area using CBCT rather than on canal fillings, these procedures are unlikely to have influenced the analyzed measurements. Although the crown was removed and simulation of the periodontal ligament and lamina dura was not performed, this in vitro model was selected to standardize the visualization of the furcal perforation and control imaging variables. Nonetheless, the absence of structures simulating soft and cortical tissues may reduce the translational potential of the findings, as noted in previous studies.^4,32^ A pilot study standardized procedures, including specimen selection based on pulp chamber floor dimensions, parallax adjustments, reference point determination, and simultaneous measurements using both a micrometer and e-Vol DX software.^20-22,32^

The application of BAR filters was tailored to the density and atomic number of each sealing material, ensuring optimal artifact reduction without compromising measurement accuracy. The results highlight the clinical utility of the BAR algorithm in endodontics, as the reduction of blooming artifacts enhances the accuracy of CBCT-based measurements, improving diagnostic precision, treatment planning, and long-term outcomes. By mitigating the impact of high-density materials, the BAR algorithm supports the detection of furcal perforations, cracks, and fractures, as well as the evaluation of sealing material adaptation.^20-22,32^

The absence of a non-perforated control group limits the interpretation of baseline image characteristics. Future studies should incorporate negative controls to isolate the impact of the sealing materials and perforation geometry on CBCT measurements. This study was conducted in vitro, not entirely replicating the complexity of clinical conditions, such as soft tissue attenuation or motion artifacts. Furthermore, only one CBCT scanner and a single voxel size were evaluated. Future studies should include other CBCT systems, different scanning parameters, and various artifact reduction algorithms, thus allowing for generalization of the findings. Although this study focused on specific sealing materials, the findings suggest broader applicability to other high-density endodontic materials. Future studies should evaluate the BAR algorithm across different CBCT systems, voxel sizes, and imaging protocols to confirm its generalizability. Comparisons with other artifact reduction algorithms, such as MAR, could provide further insights into optimizing CBCT imaging for complex cases. The BAR algorithm in the e-Vol DX software proved effective in reducing blooming artifacts without altering the dimensional measurements of sealing materials. These findings demonstrate the potential of the algorithm to enhance diagnostic accuracy and treatment predictability in endodontics, particularly for challenging cases involving high-density materials, and emphasize the importance of selecting appropriate sealing materials and imaging protocols to enhance diagnostic accuracy and treatment outcomes in cases of furcal perforation. While the study demonstrated consistency of the BAR algorithm across materials, the lack of measurements without artifact reduction precludes direct comparative evaluation. The results contribute to ongoing advancements in CBCT imaging, supporting improved clinical outcomes and long-term tooth preservation.^6,7,9,13-15,19,20,24-26,33-38^

Radiologists should be encouraged to include quantitative assessments of repair material dimensions in their reports, particularly in complex cases involving perforation repairs. The BAR algorithm may be integrated into standardized diagnostic protocols to ensure accuracy in follow-up assessments and reintervention planning.

## Conclusion

The findings of this ex vivo observational study indicate that the BAR algorithm integrated into the e-Vol DX software effectively minimizes metallic artifacts generated by radiopaque sealing materials — such as Biodentine, MTA ProRoot, MTAFlow, and amalgam — in CBCT imaging. Additionally, the BAR algorithm provided measurements of furcal perforation diameters with accuracy comparable to that of digital micrometry, underscoring the potential of the BAR algorithm for precise dimensional assessment in endodontic applications involving high-density materials. These outcomes reinforce the clinical applicability of the algorithm for enhancing diagnostic reliability in cases requiring detailed radiographic evaluation.

## Data Availability

The authors declare that all data generated or analyzed during this study are included in this published article.
